# Oncological Treatment-Related Fatigue in Oncogeriatrics: A Scoping Review

**DOI:** 10.3390/cancers14102470

**Published:** 2022-05-17

**Authors:** Louise André, Gabriel Antherieu, Amélie Boinet, Judith Bret, Thomas Gilbert, Rabia Boulahssass, Claire Falandry

**Affiliations:** 1 Hospices Civils de Lyon, Geriatrics Department, Hôpital Lyon Sud, 69230 Saint Genis-Laval, France; louise.andre@chu-lyon.fr (L.A.); gabriel.antherieu@chu-lyon.fr (G.A.); amelie.boinet@etu.univ-lille.fr (A.B.); judith.bret@chu-lyon.fr (J.B.); thomas.gilbert@chu-lyon.fr (T.G.); 2 Research on Healthcare Professionals and Performance RESHAPE, Inserm U1290, Lyon 1 University, 69008 Lyon, France; 3 Geriatric Coordination Unit for Geriatric Oncology (UCOG) PACA Est CHU de Nice, 06000 Nice, France; boulahssass.r@chu-nice.fr; 4 FHU OncoAge, 06000 Nice, France; 5 Faculty of Medicine, University of Nice Sofia Antilpolis, 06000 Nice, France; 6 CarMeN Laboratory, INSERM U.1060/Université Lyon1/INRA U. 1397/INSA Lyon/Hospices Civils Lyon, Bâtiment CENS-ELI 2D, Hôpital Lyon Sud Secteur 2, 69310 Pierre-Bénite, France; 7 UCOGIR—Auvergne-Rhône-Alpes Ouest–Guyane, Hôpital Lyon Sud, 69495 Pierre-Bénite, France; 8 Faculty of Medicine and Maieutics Charles Mérieux, Lyon 1 University, 69310 Pierre-Bénite, France

**Keywords:** fatigue, cancer-related fatigue, oncogeriatrics, iatrogenic, toxicity

## Abstract

**Simple Summary:**

Fatigue in older patients has multiple etiologies, as this symptom may be cancer-related, treatment-related, age-related, or part of frailty syndrome. Physicians need to identify this symptom and understand its risk factors but also evaluate the risk/benefit ratio of cancer treatments considering the risk of impairing the patient’s quality of life. This scoping review was aimed to present the level of information currently available on any-grade fatigue and grade 3 or more fatigue for each cancer treatment regimen, either in general or in older populations, for the most prevalent tumors.

**Abstract:**

Fatigue is a highly prevalent symptom in both cancer patients and the older population, and it contributes to quality-of-life impairment. Cancer treatment-related fatigue should thus be included in the risk/benefit assessment when introducing any treatment, but tools are lacking to a priori estimate such risk. This scoping review was designed to report the current evidence regarding the frequency of fatigue for the different treatment regimens proposed for the main cancer indications, with a specific focus on age-specific data, for the following tumors: breast, ovary, prostate, urothelium, colon, lung and lymphoma. Fatigue was most frequently reported using the National Cancer Institute Common Terminology Criteria for Adverse Events (NCI CTCAE) versions 3 to 5. A total of 324 regimens were analyzed; data on fatigue were available for 217 (67%) of them, and data specific to older patients were available for 35 (11%) of them; recent pivotal trials have generally reported more fatigue grades than older studies, illustrating increasing concern over time. This scoping review presents an easy-to-understand summary that is expected to provide helpful information for shared decisions with patients regarding the anticipation and prevention of fatigue during each cancer treatment.

## 1. Introduction

**Figure 1:**
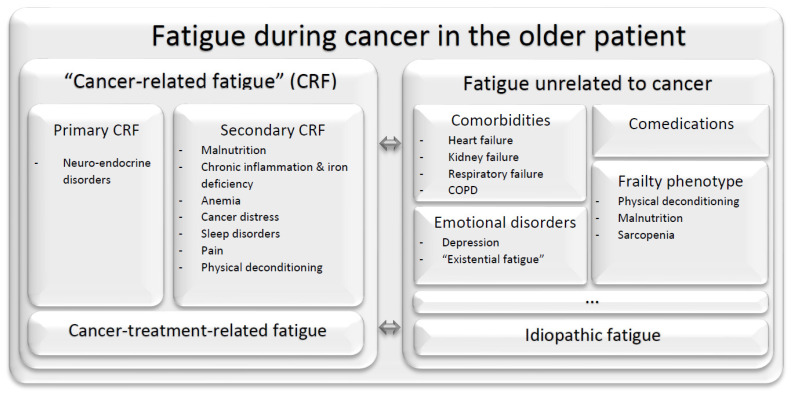
The multiple etiologies of fatigue in older patients with cancer. Abbreviations: CRF: cancer-related fatigue; COPD: chronic obstructive pulmonary disease.

Fatigue is defined as a persistent sense of physical, emotional, and/or cognitive tiredness or exhaustion that is not proportional to recent activity and that interferes with usual functioning [1]. It is highly prevalent both in older people, in whom it is strongly associated with negative health-related events [2], and during cancer and its treatment [3]. The understanding of fatigue is hampered by several issues, including the difficulty for objective evaluation [4] and the variability in the methods used to collect relevant data on fatigue (note the coexistence of the “fatigue” or “fatigability”, “asthenia”, “weakness”, “lethargy” terms and different definitions of this concept) that has led to a wide range of estimates of its frequency [5,6], as well as its complex pathophysiological mechanisms [7]. Fatigue in older patients with cancer is multicausal (Figure 1); it may be interpreted as a primary or secondary cancer-related fatigue and may be related to one (or more) of several comorbidities and comedications, but it may also be related to emotional disorders; it may also be considered to be a syndrome by itself—an age-related nosological entity called “idiopathic fatigue” according to the fifth version of the Diagnostic and Statistical Manual of Mental Disorders (DSM-5) [8]; finally, it is one of the pillars of the frailty phenotype [9]. Consequently, 70 to 80% of oncogeriatric patients questioned specifically on the topic report fatigue, and this has a severe impact on the quality of life [10,11,12]. However, fatigue has historically been seldom presented as an expected side effect of cancer treatments or included in the assessment of the risk/benefit ratio during cancer management.

With the development of targeted therapies and their related long-lasting fatigue, the consideration of fatigue has changed; during the last two decades, fatigue has been demonstrated as the most prevalent symptom in oncology [7,13], affecting both activities of daily living (ADL) and social life, interfering with thymia and cognition, predicting prognosis [14], and (above all) acting as a powerful differentiation criterion for therapeutics that has led to a change of mindset, illustrated by the increasing focus on patient-reported outcomes, quality of life, and shared decisions [15].

A significant step was taken in 2009 with the release of fourth version of the National Cancer Institute Common Terminology Criteria for Adverse Events (CTCAE) that standardized fatigue with the following grading [16]: (1). Fatigue relieved by rest; (2). fatigue not relieved by rest, limiting instrumental ADL (IADL); (3). fatigue not relieved by rest, limiting self-care ADL; and no grade 4 when the third version [17] was difficult to interpret/to quote. Future editions changed the grading to: (1). Mild fatigue over baseline; (2). moderate or causing difficulty performing some ADL; (3). severe fatigue interfering with ADL; and (4). disabling. It should be noted that the release of the fifth version in 2017 did not modify this scale [18], which has integrated the standardization of ADL and IADL scoring in parallel with its everyday use in oncogeriatric care.

Moreover, much work has been undertaken to decipher fatigue and its modifiable risk factors. Anemia and non-anemic iron deficiency contribute to fatigue [19,20] and fatigability [21] and accordingly should be tracked; malnutrition is associated with fatigue and decreased quality of life, cancer distress and cognitive impairment contribute to the psychosomatic signs of fatigue, and sedentary lifestyle and sarcopenia contribute to physical deconditioning, in turn leading to fatigability and fatigue. All these modifiable risk factors have led to the development of standardized work-up and the development of prehabilitation strategies, focusing on limiting and postponing the symptoms of fatigue.

Is this context, it appeared necessary to report the current level of evidence on the frequency of fatigue occurrence for the different treatment regimens proposed for the main cancer indications, with a specific focus on data specific to older adults, as has been done previously for other frequent treatment-related adverse events such as febrile neutropenia [22] and emesis [23].

## 2. Materials and Methods

To be included in the review, studies needed to focus on the treatment regimen currently proposed for the most frequent neoplasias (breast, ovary, non-Hodgkin’s lymphoma, prostate, lung, colon, and urothelial), reporting or not fatigue frequency of occurrence, with data specific to older adults present or absent. The inclusion criteria were that there were original studies that were published in the English or French language. To identify relevant studies, PubMed/MEDLINE was searched until December 2021. The search strategy used a combination of MeSH terms (including “fatigue” and “asthenia”), as well as “lethargy” and “weakness”. Additional manuscripts, not retrieved using electronic search strategy, were also included in the eligibility screening process by the authors. The final search results were exported into ZOTERO v6.0.4 (Digital Scholar, Vienna, VA, USA), and duplicates were removed. Titles/abstracts were independently evaluated for eligibility by two reviewers (either L.A. and C.F. or G.A. and C.F.). Any disagreement between reviewers was resolved through discussion. The reviewers assessed the full texts of articles retained after the title/abstract screening. Considering the exclusion of scoping reviews from PROSPERO registration [9], this analysis was not registered on any registration platform.

In the first step of data analysis, reviewers examined the presence or not of information on fatigue and data specific to older adults; treatment protocols were semi-quantitatively classified according to the frequency of grade 3 fatigue as defined by the CTCAE classification.

## 3. Results

**Figure 2:**
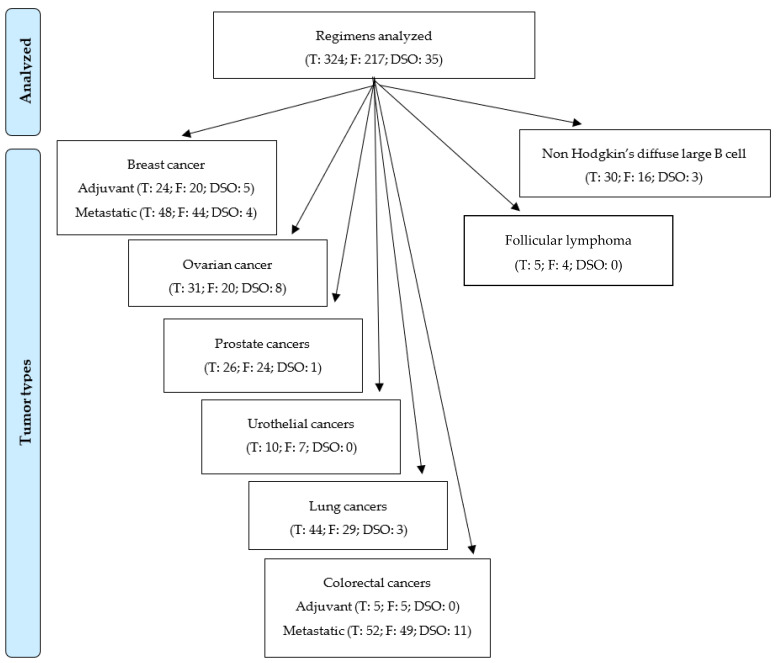
Flowchart detailing for each cancer type, indicating the total (T) number of regimens analyzed, the number of regimens for which fatigue (F) was reported, and the number including data specific to older adults (DSO).

A total of 324 regimens were included; data on fatigue were available for 217 (67%) of these, and fatigue data specific to older patients were available for 35 (11%; Figure 2). There were 24 breast cancer adjuvant treatment regimens, of which 20 (83%) reported data on fatigue (F) and 5 (21%) reported data specific to older patients (DSO); 48 breast cancer metastatic treatment regimens (F: 43 [93%]; DSO: 4 [9%]); 31 ovarian cancer treatment regimens (F: 20 [65%]; DSO: 8 [26%]); 26 prostate cancer treatment regimens (F: 24 [92%]; DSO: 1 [4%]); 10 urothelial cancer treatment regimens (F: 7 [70%]; DSO: 0 [0%]); 44 lung cancer treatment regimens (F: 29 [66%]; DSO: 3 [7%]); 5 colorectal cancer adjuvant treatment regimens (F: 5 [100%]; DSO: 0 [0%]); 52 colorectal cancer metastatic treatment regimens (F: 49 [94%]; DSO: 11 [21%]); 30 non-Hodgkin’s diffuse large B cell lymphoma treatment regimens (F: 16 [53%]; DSO: 3 [10%]); and 5 follicular lymphoma treatment regimens (F: 4 [80%]; DSO: 0 [0%]).

### 3.1. Fatigue during Breast Cancer Treatment

Fatigue during and after localized breast cancer treatment has been a large matter of debate. In a meta-analysis published in 2016, Abrahams et al. identified 27 studies and a total of 12,237 patients treated in trials evaluating the frequency of fatigue occurrence and risk factors of severe fatigue in breast cancer survivors. The tools used for fatigue assessment were highly heterogeneous, sometimes with several different thresholds for the same tool. The overall frequency of fatigue was 27% with a wide range of values (7 to 55%) and a large decrease in this frequency in the first 6 months after treatment completion. The strongest risk factor for severe fatigue was an association of surgery, radiotherapy, chemotherapy and hormonal treatment (RR: 1.38; 95% CI: 1.15–1.66); this was followed by the association of surgery, radiotherapy and chemotherapy (RR: 1.18; 95% CI: 1.05–1.33); surgery and radiotherapy (RR: 0.87; 95% CI: 0.78–0.96); and surgery only (RR: 0.83; 95% CI: 0.70–0.98) [5]. In another study reported by Schmidt et al., the determinants and correlates of physical, affective and cognitive fatigue assessed with the 20-item Fatigue Assessment Questionnaire included obesity (physical), poor social support and worries about the future (affective), poor sleep quality and previous use of psychopharmaceuticals (physical, affective, and cognitive) [24]. In addition, the currently ongoing CANTO longitudinal cohort has led to the validation of a predictive model of severe fatigue; severe fatigue was defined as a fatigue score ≥40% according to the European Organisation for Research and Treatment of Cancer quality-of-life core questionnaire with 30 items (EORTC QLQ-C30). In this model, retained risk factors for severe fatigue at 2 years after diagnosis were pretreatment fatigue, younger age, higher body mass index, current smoking behavior, worse anxiety, insomnia, and pain at diagnosis; hormonal treatment was found to be a risk factor for severe fatigue 4 years after diagnosis [25].

In the adjuvant setting, 34 regimens were identified; data on fatigue (any-grade and/or grade 3 or more) were reported for 25 of these, and data specific to older patients were reported for 3; 32 concerned chemotherapies with or without targeted treatment regimens, and 2 concerned adjuvant hormonal treatments (Table 1). In the chemotherapy with or without targeted treatment subgroup, any-grade fatigue rates (when reported) were high, ranging from 49 to 77% for the AC regimen (adriamycine and cyclophosphamide), to 81% for the TAC regimen (docetaxel, adriamycine, and cyclophosphamide). When considering grade 3 or more fatigue, data were more frequently available than any-grade fatigue; there were very low rates (1–3%) for adjuvant capecitabine after neoadjuvant chemotherapy [26], docetaxel cyclophosphamide [27] and paclitaxel trastuzumab [28], but rates reached more than 20% for the AC → docetaxel sequential regimen (22%) and dose-dense adriamycine docetaxel (ddAT; 28%). Reinisch et al. evaluated the impact of age on the tolerance of three regimens, A(E)C-[T/P], ddAT and TAC; there was a trend towards more frequent grade 3 or more fatigue in older subgroups [29]. When considering hormonal treatment and in contrast to the previously described analyses on chronic fatigue in breast cancer survivors and the impact of hormonal treatments on such chronic fatigue [5], the reported rates of any-grade fatigue and grade 3 or above fatigue were overall low (respectively, from 3 to 18% and 1% for tamoxifen [30,31]; from 1 to 16% and 0% for anastrozole [31,32]; 30% and 1% for letrozole [33] with a higher frequency of any-grade fatigue [45%] in study conducted specifically on older patients [34]; and 24% and 1% for exemestane [35]).

In the metastatic setting, 48 regimens were identified; data on fatigue (any-grade and/or grade 3 or more) were reported in all but 4 of them; 32 concerned chemotherapies with or without targeted treatment regimens or antibody–drug conjugates, 5 hormonal treatments alone, 9 hormonal treatments associated with targeted therapies and 2 targeted therapies only. In the chemotherapy with or without targeted treatment subgroup of regimens, any-grade fatigue rates (when reported) were highly variable, ranging from 10% for ixabepilone to up to 35 to 54% for eribulin and 60% for metronomic docetaxel capecitabine—two regimens proposed in late lines of treatments. Data were more frequently available for grade 3 or more fatigue than for any-grade fatigue; rates were very low (2%) for pertuzumab + trastuzumab + paclitaxel or nab-paclitaxel (PERUSE study [82]) and trastuzumab + docetaxel (CLEOPATRA study control arm [76]), but they reached more than 20% for 100 mg/m^2^ of docetaxel (15 to 24%) and gemcitabine vinorelbine (24%); in the metastatic context and according to the 5th advanced breast cancer conference guidelines, this highlights the need to favor monotherapy in such patients [101], and, when considering docetaxel, to prefer a 3w-75 mg/m^2^ schedule. For hormonal treatments alone, the rates of any-grade fatigue varied greatly between studies; “lethargy” was estimated to be 1% for anastrozole and 3% for tamoxifen in older reports [32], and it reached 16 and 18%, respectively, in more recent studies [31]; from 11 to 27% for letrozole [30,31,83,84]; from 22 to 26% for exemestane [31,85]; and from 31 to 44 % for fulvestrant [86,87,88,89,90]. A high number of these values were recently estimated, since such regimens were control arms of the recent studies on CDK4–CDK6 inhibitors. The rates of grade 3 or more fatigue with hormonal treatments alone were very low and probably close to any control population (0 to 3%). The addition of CDK4–CDK6 inhibitors is more frequently associated with any-grade and grade 3 or more fatigue. A similar impact was observed for the addition of everolimus to exemestane (23–50% any-grade fatigue compared to 22–26% with exemestane only and from 2 to 4% grade 3 or more fatigue compared to 0%) and for the addition of lapatinib to letrozole (21 compared to 17% any-grade fatigue and 2% compared to <1% grade 3 or more fatigue in the only comparative study [91]). Finally, the use of poly (ADP-ribose) polymerase inhibitors (PARPi) was associated with a higher frequency of any-grade and grade 3 or more fatigue compared to placebo in patients who had previously received several lines of treatment and in whom fatigue was frequent, including those in the placebo arm. Data specific to older patients were reported for 3/48 these regimens; the DOGMES study was a phase 2 study that evaluated the feasibility of pegylated liposomal doxorubicin in first-line metastatic breast cancer aged ≥70 years and reported a very high rate of any-grade and grade 3 or more fatigue (69% and 22%, respectively [66]); the BALLET subgroup analysis according to age (<70 years vs. ≥70 years old) of the exemestane–everolimus study found (non-comparative) higher rates of any-grade fatigue (69% vs. 61%, respectively) and a doubling of grade 3 or more fatigue (6% vs. 3%, respectively) [94]; finally, a recent pooled study of the PALOMA-1, -2 and -3 studies with a subgroup analysis on (selected) older patients (<65 years, 65–74 years, and ≥75 years) did not find any trend related to age except for a higher frequency of grade 3 or more fatigue ≥75 years [96].

### 3.2. Fatigue during Ovarian Cancer Treatments

In contrast to breast cancer, fatigue has been historically poorly evaluated during for ovarian cancer treatments, with the risk/benefit ratio of chemotherapy considered to be largely beneficial, at least as first-line treatment. Again, in contrast to breast cancer treatments, the impact of age on cancer treatments has been by far more explored. In addition, there is greater awareness of the management of targeted treatments in older patients with this disease that is shared by both clinicians and the pharmaceutical industry, which is well-illustrated by the publication in 2019 of a review paper of the Young International Society of Geriatric Oncology (SIOG) reporting several subgroup analyses of pivotal trials according to age; nevertheless, the authors highlighted that these populations remain selected and appealed for specific trials to be conducted in older patients [102].

Considering first-line chemotherapy, 17 different regimens were identified, including the maintenance regimens with bevacizumab with or without PARPi (Table 2). Data on any-grade fatigue were available for only eight of them, generally the most recent studies, and data for four regimens concerned the tolerance of maintenance treatments. Among these, the frequency of fatigue was generally high, between 35 and 63% for any-grade fatigue and between 1 and 5% for grade 3 or more fatigue, with high levels of remnant fatigue in control arms (any-grade and grade 3 or more fatigue in control arms of SOLO1 and PRIMA studies: 42% and 2%, respectively [103], and 30% and <1%, respectively [104]). Considering data specific to older patients, six regimens were evaluated, and all of these studies reported an important impact of age and frailty, since some of them were truly specific to older patients with a special interest for vulnerable populations [105,106] on cancer-related fatigue: any-grade fatigue ranged from 38% in the MITO-5 study reported by Pignata et al., who evaluated an older-specific regimen of carboplatin AUC2 and paclitaxel for 3 of 4 weeks [107], to 85% when the same regimen was evaluated in a population with a geriatric vulnerability score (GVS) of 3 or more [106].

Considering platinum-sensitive relapse, 10 regimens were included in the analysis, and data on fatigue were available in all but 2 of them. Any-grade fatigue varied from 28% to 62%, with higher rates in PARPi maintenance regimens (59% in the NOVA trial investigating niraparib maintenance [121], 62% in the SOLO2 trial investigating olaparib maintenance [120], and 69% in ARIEL3 trial investigating rucaparib maintenance [134]), in comparison to chemotherapy-only regimens (41%, 44%, and 37%, respectively, in the control arms of these trials) and targeted treatment-only regimens (39% and 40%, respectively, for the niraparib only and the niraparib + bevacizumab arms of the AVANOVA2 trial [124]). Grade 3 or more fatigue rates varied from 2% to 8%, with a higher (non-comparative) impact versus placebo reported with niraparib maintenance (8% vs. 1% in the placebo arm [121]) rucaparib maintenance (7% vs. 3%, respectively [134]), and olaparib maintenance (4% vs. 2%, respectively [120]).

Considering platinum-resistant relapse, eight regimens were identified; data on fatigue were reported in four of them and were highly variable between studies, both for any-grade fatigue (22–44% for pegylated liposomal doxorubicin, 31–46% for 3w-topotecan (5-day course), and 32% for w-topotecan) and for grade 3 or more fatigue (0–2% and 2–22% for 3-weekly [126,127] and weekly [128,129] topotecan regimens, respectively; 11% for gemcitabine [130]; and 1–6% for pegylated liposomal doxorubicin [117,130,131]). Of note, the addition of bevacizumab to chemotherapy in the AURELIA trial was associated with (non-comparative) lower levels of grade 3 or more fatigue than chemotherapy alone (4% vs. 10%, respectively [133]), illustrating the important role of tumor-related symptoms in complaints of fatigue. No data specific to older patients were reported in this setting.

### 3.3. Fatigue during Prostate Cancer Treatments

During the development of novel hormonal therapies for prostate cancer, first in the castration-resistant metastatic setting and then in the castration-sensitive metastatic setting and even in the non-metastatic castration-resistant setting, the preservation of quality of life, treatment-related cognitive symptoms, and fatigue were factors used to differentiate the tolerance of abiraterone acetate and other next-generation androgen receptor inhibitors (such as enzalutamide, apalutamide, and darolutamide; see Table 3). Another point is the putative differential impact of LHRH agonists versus LHRH antagonist (degarelix) on castration-related fatigue [135]. This is of importance because castration-related fatigue appears to have a high impact on the quality of life, as found in a 2013 systematic review published by Langston et al., who reported that the frequency of any fatigue and severe fatigue reached 74% and 14%, respectively [6].

In the non-metastatic castration-resistant setting, the addition of apalutamide to androgen-deprivation therapy (ADT) was found to more than double the respective rates of any-grade fatigue (33% vs. 14%) and grade 3 or more fatigue (3% vs. 1%) [136].

In the metastatic castration-sensitive setting, and with the exclusion of the meta-analysis conducted by Langston et al. [6] who did not use the CTCAE grading system, the frequency of any-grade fatigue associated with degarelix was 3% (arm with 240 mg at induction/80 mg at maintenance) vs. 6% for leuprolide in a 12-month randomized study [135]. In the control arms of more recent studies (LATITUDE, STAMPEDE, CHAARTED, TITAN, and SPARTAN), however, any-grade and grade 3 or more fatigue ranged from 14 to 20% and from <1 to 4%, respectively [136,137,138,139,140,141]. Concerning the impact of next-generation antiandrogens, abiraterone acetate (an androgen synthesis inhibitor) weakly impacted fatigue rates versus ADT only; the rates of any-grade and grade 3 or more fatigue were, respectively, 14% vs. 13% and 2% vs. 2% [137,138]. Conversely, next-generation androgen receptor inhibitors induced an increase in any-grade and grade 3 or more fatigue by 4% and 1 to 4%, respectively; for enzalutamide [139,140]; and from 5 to 15 and <1 to 1%, respectively, for apalutamide [136,141]. Finally, the addition of docetaxel 75 mg/m^2^/3w led to significantly higher frequency of any-grade fatigue from 20% to 74% and grade 3 or more fatigue from 1–4% to 4–7% [145,146].

In the metastatic castration-resistant setting, the first indication in which next-generation antiandrogens were historically developed, abiraterone acetate poorly impacted fatigue rates compared to ADT only, as in the castration-sensitive setting, but the basal rates were probably due to the cancer itself: any-grade fatigue rates reached 44% (vs. 43%) after docetaxel (COU-AA-301 study) and 39% (vs. 34%) in chemo-naive patients (COU-AA-302 study) [147,149]. Conversely, enzalutamide induced an increase in any-grade fatigue by 10% (36% vs. 26%) in chemo-naïve patients [153] and by 5% (34% vs. 29%) after docetaxel [151], with a weak impact on grade 3 or more fatigue rates in both settings [151,153]. Docetaxel, and to a lesser extent cabazitaxel, regimens induced high levels of any-grade and grade 3 or more fatigue. Bi-weekly and weekly docetaxel regimens did not seem to significantly reduce the frequency of fatigue, as any-grade and grade 3 or more, respectively, reached 53% and 5% of patients under the standard 3-weekly regimen [154], 49% and 5% of patients under the weekly regimen [154], and 65% and 3% of the cycles under the bi-weekly regimen [155]. The frequency of any-grade fatigue was lower with cabazitaxel (37%), even at its higher dose (25 mg/m^2^), but the frequency of grade 3 or more was the same (5%) [156]; surprisingly, the study comparing a 20 mg/m^2^ regimen to a 25 mg/m^2^ regimen did not evaluate fatigue as a significant adverse event [157]. Under Radium 223 chloride, the frequency of any-grade fatigue was strictly the same as in the control arm (24%) and the frequency of grade 3 or more fatigue was even lower (3% vs. 6%, respectively) [158]. Olaparib treatment induced higher rates of any-grade fatigue than the physician’s choice of enzalutamide or abiraterone (control) (41% vs. 32%, respectively) but lower rates of grade 3 or more (3% vs. 5%, respectively) [159].

### 3.4. Fatigue during Urothelial Cancer Treatments

When considering urothelial cancers, data on fatigue occurrence under standard treatment were sparse (Table 3). Among first-line treatments, the classical MVAC induced a very high rate of grade 3 or more fatigue (24%), far higher than the paclitaxel 225 mg/m^2^/carboplatin AUC6 regimen (10%) [160], but no data were reported on high-dose intensity MVAC, cisplatin–gemcitabine and carboplatin–gemcitabine regimens; avelumab maintenance induced an increase in any-grade fatigue by 11% and in grade 3 or more by 1% compared to the control (placebo) arm [164]. In the second line, contrariwise, pembrolizumab monotherapy was associated with a decreased rate of any-grade and grade 3 or more fatigue compared to the control (chemotherapy) arm (14% vs. 28% and 1% vs. 4%, respectively) [166]; vinflunine was associated with a high prevalence of any-grade fatigue that was, however, lower than in the control arm (50% vs. 61%, respectively), illustrating the high impact of tumor-related fatigue [165]. In the third line, enfortumab vedotin increased any-grade fatigue by 8% and grade 3 or more fatigue by 2% compared to the control (chemotherapy) arm [168].

### 3.5. Fatigue during Colorectal Cancer Treatments

Contrary to other tumor models, fatigue has been assessed in studies investigating colorectal cancer for many years in both adjuvant and metastatic settings (Table 4). In the adjuvant setting, data on any-grade and grade 3 or more fatigue were available for the 5 currently used regimens; in the metastatic setting, they were available for all but one of the 35 regimens reported, and data specific to older adults were available for 8 of the latter. In the adjuvant setting, recent studies from the International Duration Evaluation of Adjuvant Chemotherapy (IDEA) collaboration have shed light on the high impact of chemotherapy duration on cumulative adverse events, including fatigue: the SCOT, TOSCA and IDEA studies reported higher rates of both any-grade fatigue (39 to 90% vs. 28 to 86%) of grade 3 or more fatigue (4 to 8% vs. 1 to 8%) in 6 vs. 3 arms of treatment, respectively [169,170,171]. In the MOSAIC adjuvant pivotal study, any-grade fatigue reached 63% in the FOLFOX arm compared to 25% in the 5-FU/leucovorin arm, demonstrating a high impact of the addition of oxaliplatin on fatigue in parallel to the classical impact on peripherical neuropathy. In most studies in the adjuvant setting, grade 3 or more fatigue was reported less than 5% [172].

In the metastatic setting, however, reported rates of severe fatigue with the oxaliplatin and 5-FU derivatives (5-FU/leucovorin or capecitabine) reached almost 10% and even higher in older populations (16% in the study reported by Feliu et al., who included patients aged ≥70 years [182], and 22% in the study reported by Twelves et al., who included patients aged ≥65 years [181]). Notably, in the FOCUS2 trial on older/frail patients who were not candidates for standard full-dose chemotherapy, Seymour et al. evaluated the impact of both the addition of oxaliplatin and the replacement of fluorouracil with capecitabine on treatment efficacy and tolerance in a 2 × 2 factorial trial, with four regimens at 80% of the standard full dose (FU: levofolinate 175 mg 2-h intravenous infusion, fluorouracil 320 mg/m^2^ 5-min intravenous bolus, and fluorouracil 2240 mg/m^2^ 46-h intravenous infusion; Cap: capecitabine 1000 mg/m^2^ orally twice per day on days 1–15 of each 21-day cycle; OxFU: previous FU regimen with the addition of oxaliplatin 68 mg/m^2^ by 2-h intravenous infusion concurrent to levofolinate; CapOx: previous Cap regimen with the addition of oxaliplatin 104 mg/m^2^ 2-h intravenous infusion every day 1 of each 21-day cycle); oxaliplatin addition had no impact on the frequency of grade 3 or more lethargy (10% vs. 12%, respectively; *p* = 0.63), but a trend towards a higher frequency was found with the replacement of FU by capecitabine (8% vs. 14%, respectively; *p* = 0.06) [184]. When considering raltitrexed as an alternative to a 5-FU derivative, either alone or in association with oxaliplatin (TOMOX), the frequencies of any-grade and grade 3 or more fatigue were similar to their 5-FU-based counterparts. The addition of bevacizumab to the 5-FU derivative ± oxaliplatin did not meaningfully alter fatigue rates of FOLFOX [177,209,212] and XELOX [213,214,215]. When considering the other classical association of a 5-FU derivative with irinotecan, FOLFIRI and CAPIRI/XELIRI induced similar rates of fatigue, as the rates of grade 3 or more fatigue varied from 0 and 8% [185,186,187,188,189,190,191,192,193,194,195]. By contrast, the addition of either bevacizumab or aflibercept seemed to be associated with a higher frequency of grade 3 or more fatigue (1–9% for FOLFIRI–bevacizumab [189,207,208,209], 7–17% for XELIRI–bevacizumab [210,211], and 17% for FOLFIRI–aflibercept [217]). Data regarding the impact of cetuximab and panitumumab were highly heterogeneous, probably due to the different lines of treatment studied; one study demonstrated a high frequency of grade 3 or more fatigue with cetuximab alone in later lines (33% vs. 26% in the control arm [218]), but studies on cetuximab in association with either FOLFOX, XELOX, FOLFIRI, XELIRI, or irinotecan alone reported relatively low frequencies of grade 3 or more fatigue [188,191,208,219,220,222]. Similarly, the frequencies of fatigue with chemotherapy triplets associated with 5-FU derivatives, irinotecan and oxaliplatin, were highly variable; rates of grade 3 or more fatigue were relatively low (3 to 6%) in studies investigating FOLFOXIRI/FOLFIRINOX [186,189] and XELOXIRI [199] but reached 12% for bevacizumab–FOLFOXIRI [189,207] and even 32% for cetuximab–FOLFIRINOX [221].

When considering the trifluridine–tipiracil (TAS-102) ± bevacizumab, the frequencies of fatigue were also heterogeneous; any-grade fatigue ranged from 24 and 85%, illustrating the high level of chronic fatigue, independently of the treatment, in later lines [227,228,229]. In a pivotal trial reported by Mayer et al., the frequencies of any-grade and grade 3 or more fatigue were, respectively, 35% and 4% vs. 23% and 6% in the placebo arm [227]; in a comparative phase II trial reported by Pfeiffer et al. evaluating the addition of bevacizumab, any-grade fatigue frequencies were exactly the same in both arms (85%), and the grade 3 or more fatigue frequency was 11% in the TAS-102 arm and 7% in the TAS-102 + bevacizumab arm [228].

In the field of targeted therapies, beyond bevacizumab and cetuximab, regorafenib was associated in later lines with a manageable tolerance, including fatigue, in the CORRECT pivotal trial [225]; however, the REGOLD study demonstrated very high levels of fatigue in older patients (90% any-grade and 45% grade 3 or more fatigue), leading the authors to advise precautious conditions of follow-up for in the oldest patients (≥80 years) [226]. In BRAF V600E-mutated patients, the BEACON study demonstrated a significant advantage for the association of encorafenib and cetuximab over usual care, with an acceptable tolerance profile—in particular considering fatigue (any-grade and grade 3 or more fatigue rates of 30% and 4% vs. 27% and 4%, respectively); future specific studies are needed to evaluate the impact of age on these frequencies [230]. Recent years were also marked by the advent of immunotherapy in cancers with microsatellite instability (MSI+). In the KEYNOTE 177 study investigating pembrolizumab monotherapy as a first-line treatment compared to usual care, the frequencies of any-grade and grade 3 or more fatigue were lower in the immunotherapy arm (38% and 6%, respectively) than in the chemotherapy arm (50% and 9%, respectively) [231].

### 3.6. Fatigue during Lung Cancer Treatments

In the context of non-small cell lung cancer, a shift in the assessment of fatigue was observed with the advent of modern treatments—firstly targeted therapies and then immunotherapies—in parallel with the major impact that these had on overall survival rates (Table 5). Classical chemotherapies are generally delivered at higher dose intensities than in other solid tumor models, thus explaining higher rates of any-grade and grade 3 or more fatigue, with the latter frequently exceeding 10%. However, there have been some disparities; for instance, the frequency of fatigue associated with carboplatine–paclitaxel (with paclitaxel at 225 mg/m^2^) was historically between 9% and 15% [232,233], whereas it ranged from 3 to 5% for carboplatine–paclitaxel associated with bevacizumab [234,235] and was 3% for carboplatine–paclitaxel associated with bevacizumab and atezolizumab (with standardized doses of carboplatine AUC6 and paclitaxel 200 mg:m^2^ every 3 weeks) [235]. As in the breast or ovarian cancer contexts, it is notable that tyrosine kinase inhibitors, classically associated with frequent chronic fatigue, induce moderate-to-high frequencies of any-grade fatigue (from 27% for crizotinib to 47% for ceritinib) but relatively low frequencies of grade 3 or more fatigue (from 0% for erlotinib to 5% for ceritinib). When considering targeted therapies in association with chemotherapy, the addition of bevacizumab has not, as in other tumor contexts and discussed above, led to an increase in the frequency of any-grade fatigue. Conversely, immunotherapies such as pembrolizumab [236], and nivolumab [237] appear to have increased the occurrence of fatigue, except for atezolizumab, as indicated above [235]. Future studies will probably improve our knowledge on such interactions.

Considering small cell tumors and given the age of the majority of available trials, potentially as well as the poor prognosis of the disease, data on fatigue have been sparse and limited to the most recent studies. Of the 26 regimens studied, data on fatigue were reported in half of them (*n* = 13), and data specific to older adults were reported for 1 only [268]. When reported, the frequencies of any-grade and grade 3 or more fatigue were highly variable, probably due to the poor condition of patients in such tumor contexts and to the positive impact of chemotherapy, at least during the first months of treatment.

### 3.7. Fatigue during Treatments for Non-Hodgkin’s and Follicular Lymphoma

As in many other tumor models, such as small cell cancer, there has been a shift from the oldest chemotherapy regimens to the most modern ones regarding the consideration of fatigue in pivotal trials (Table 6). This was well-illustrated by the advent of rituximab in association with previous chemotherapy regimens, leading to the better reporting of fatigue in the trials [277,278,279]. In fact, no data regarding fatigue are available in papers reporting the CHOP regimen [267], even when dedicated to older patients [280,281,282]. All but one trial specifically dedicated to older patients receiving R-CHOP-based regimens did not provide data regarding fatigue [283]; one study on patients aged ≥60 years reported 49% any-grade fatigue and 0% grade 3 or more fatigue with the R-CHOP regimen [284]. Considering other chemotherapy regimens, the frequency of any-grade fatigue was moderate-to-high but with relatively low levels of grade 3 or more fatigue, with the exception of R-DHAP (9%) and R-COMP in older patients (7%) [284]. R-CVP was associated with moderate any-grade fatigue rates (53%) and almost no grade 3 or more fatigue (<1%) in the overall population but much more frequent grade 3 or more fatigue in the older patients (47 %) [285], calling into question its use in this specific population.

Considering targeted treatments, lenalinomide was associated with relatively elevated rates of grade 3 or more fatigue (7 to 9%), particularly in association with rituximab (13 to 23%) [301,302,303]. For CAR-T cells, there was a moderate-to-high frequency of any-grade fatigue, but the frequency of grade 3 or more fatigue was limited (from 1% for lisocabtagene maraleucel [307] to 6% for tisagenlecleucel [308]). No data specific to older adults were available in these first trials. A trial investigating lisocabtagene maraleucel was the only one including older patients (from 18 to 86 years old, including 10% of ≥75 years old), and fatigue rates were similar to those reported for axicabtagene ciloleucel [307,308]. Two studies reported real-life experience with commercial CAR-T, though unfortunately neither of them provided data regarding fatigue [309,310].

## 4. Discussion

This scoping review is, to our knowledge, the first to provide oncologists and oncogeriatricians with a simple and easy-to-understand summary of the level of information available on fatigue, either in general or in older populations, associated with the different treatment regimens proposed for the most prevalent tumors. Such information may enrich the shared decision process of patients and their families, as well as induce guided intervention to prevent fatigue occurrence as soon as care programs are established.

From a historical point of view, this work highlights the continuous increase in fatigue assessment in relationship with the evolution of patients’ expectations regarding a better consideration of their quality of life. This point is even more important in the oncogeriatric context, when overall survival is compromised due to cancer or other causes of death. It additionally highlights that this consideration is also dependent on the tumor model, the setting, and the modality of treatment. Notably, a higher consideration was observed for maintenance or continuous treatments compared to treatments of a shorter duration, such as adjuvant treatment for breast cancers and colon cancers. The concept of cancer survivorship, notably in the context of breast cancer, has shed light on the importance of long-lasting fatigue after chemotherapy cessation in general, but little is known on the differential impacts of individual regimens on this.

**Figure 3:**
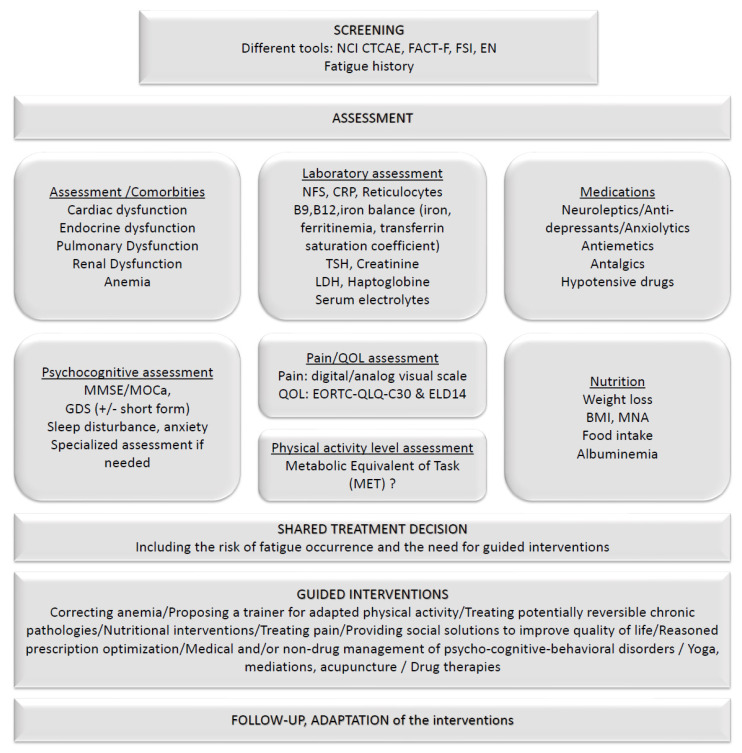
A proposed work-up on comprehensive (geriatric) assessment of fatigue.

This work has several limitations linked to its conceptualization, which led us to consider information published over a very long period during which there were changes not only in the tools used to assess fatigue (in total, 3 Versions of the NCI CTCAE: v3 to v5 [16,17,18]) but also in patients’ expectations about their quality of life, therefore leading to a higher reporting of fatigue in the most recent years. In addition, it is impossible to ensure the exhaustivity of the data; we also encourage any additional work that will, in the future, improve the quality of information and update it with the additional results of ongoing trials. Another potential limitation is the differential place of fatigue assessment in the current development of cancer treatments; here, we chose to collect data on fatigue reported as an adverse event, which was most frequently scored using the NCI CTCAE tool. It must be highlighted that several other tools are used, as well-reviewed by Langston et al. in their systematic analysis of fatigue in prostate cancer [6]; however, these tools are infrequently used, leading to difficulties in the comparability of studies and cancer treatments. Another increasingly used tool is the EORTC QLQ-C30 for quality-of-life assessment, which includes fatigue as an interviewed item, but this tool is frequently used in longitudinal studies and only relative changes have been reported [6]. Nevertheless, highlighting the high frequency of any-grade fatigue in the different tumor contexts sheds light on the need for practitioners to pay attention to this symptom that is associated with physical, thymic, and cognitive dimensions. This may also be, in an aging population of patients, a marker of pre-frailty or frailty, and, furthermore, a topic of multi-dimensional intervention. During a multidisciplinary workshop, a specific work-up was defined to help practitioners in their comprehensive (geriatric) assessment of fatigue (Figure 3).

## 5. Conclusions

This scoping review provides oncologists and oncogeriatricians a simple and easy-to-understand summary of the level of information available on any-grade fatigue and grade 3 or more fatigue for each cancer treatment regimen, either in general or in older populations, for the most prevalent tumors. Despite probably being incomplete due to the very large contours of such a review, it may provide additional information for practitioners when conducting risk/benefit ratio discussions with their patients.

## Figures and Tables

**Table 1:** Fatigue events in the most common anticancer regimens for adjuvant and advanced breast cancers.

Setting	Treatment Regimen and Reference		Fatigue Any-Grade (%)	Fatigue Gr ≥ 3 (%)
Adjuvant	AC [27,36]		49–77	2–4
	AC → docetaxel [37,38]		nr	22
	AC → paclitaxel [37]		nr	nr
	A(E)C-[T/P] [29]		nr	10
	<60		nr	9
	60–64		nr	12
	>64		nr	16
	Paclitaxel → AC [39]		nr	nr
	AT [36,40]		51	9–16
	ddAT [29,38]		nr	28
	<60		nr	25
	60–64		nr	40
	>64		nr	35
	Doxorubicin → docetaxel [41]		nr	7
	Doxorubicin/paclitaxel [42]		nr	nr
	TAC [29,43]		81	11 (asthenia)–12
	<60		nr	12
	60–64		nr	13
	>64		nr	17
	DDG epirubicin/cyclophosphamide [44]		nr	7
	Liposomal doxorubicin–cyclophosphamide ≥70 [45]		88	8
	Docetaxel/cyclophosphamide [27]		78	3
	CEF [44]		nr	6
	FAC [43,46]		71 (asthenia)	3–6
	FAC ≥ w-paclitaxel ×8 [46]		nr	8
	FAC 50 [47,48]		nr	nr
	FEC 90/100 [49,50]		nr	nr
	CMF [51,52]		nr	nr
	CMF oral [53]		nr	nr
	Doxorubicin → paclitaxel → cyclophosphamide [37]		nr	nr
	Doxorubicin docetaxel [47,48]		nr	nr
	FAC → paclitaxel [46]		nr	8
	Capecitabine [26]		26	1
	Trastuzumab/Docetaxel/Cyclophosphamide [54]		54	4
	Trastuzumab/Paclitaxel [28]		nr (gr2: 20)	2
	Trastuzumab paclitaxel for 12 weeks + trastuzumab for 9 months [28]		22	2
	Docetaxel + carboplatin + trastuzumab for 52 weeks (TCH) [41]		nr	7
	AC → TH [41]		nr	7
	Tamoxifen (ABC) [30,31]		3 (lethargy)–18	1
	Anastrozole (ABC) [31,32]		1 (lethargy)–16 (asthenia)	0
	Letrozole [33,55]		30	1
	≥70 [34]		45	nr
	Exemestane [35]		24	1
	Any adjuvant chemotherapy (CANTO) [25] Risk factors for post-treatment fatigue:		Severe fatigue (score ≥40%, EORTC QLQ-C30 fatigue item)	
	~pre-treatment fatigue ~higher BMI ~younger age ~hormonal treatment	~current smoking behavior ~worse anxiety ~insomnia ~pain at diagnosis	T1 (1 y): 36 T2 (2 y): 34 T3 (4 y): 32	
Metastatic	A → T [40]		nr	7
	AT [40]		nr	16
	Paclitaxel 175 mg/m^2^/3w [56,57]		nr	1–5
	Paclitaxel 90 mg/m^2^/3w/4 [56]		nr	5
	Paclitaxel 80 mg/m^2^/w [56]		nr	6
	Nab-paclitaxel 260 mg/m^2^/3w [57]		nr	5
	Bevacizumab paclitaxel [58,59]		nr	1–9
	Docetaxel 100 mg/m^2^/3w [60,61,62]		nr	15–24
	Docetaxel 75 mg/m^2^/3w [61]		nr	8
	Docetaxel 60 mg/m^2^/3w [61]		nr	3
	Docetaxel capecitabine [60]		nr	8
	Metronomic docetaxel capecitabine [63]		60	13
	Gemcitabine vinorelbine [64]		nr	24
	Vinorelbine [31,64]		36	17
	Liposomal Pegylated Doxorubicin [65]		nr	nr
	≥70 [66]		69	22
	Doxorubicin/vinorelbine [67]		nr	nr
	Capecitabin [68,69,70,71]		17–41	0–8
	Bevacizumab capecitabine [72]		nr	nr
	Bevacizumab capecitabine vinorelbine [72]		nr	nr
	Gemcitabine [73]		nr	nr
	Eribulin [74,75]		35–54	9
	Ixabepilone [31]		10	nr
	Trastuzumab docetaxel (CLEOPATRAc) [76]		38	2
	Trastuzumab paclitaxel [77]		8	nr
	Trastuzumab + oral vinorelbine [78,79]		42	4
	Trastuzumab vinorelbine everolimus (BOLERO-3) [79]		43	12
	Trastuzumab emsantine (EMILIAi) [80]		35	2
	Lapatinib capecitabine (EMILIAc and HER2CLIMBc) [80,81]		28–43	4
	Pertuzumab–trastuzumab–docetaxel (CLEOPATRAi and PERUSE) [76,82]		37	2–3
	Pertuzumab–trastuzumab–paclitaxel (PERUSE) [82]		nr	2
	Pertuzumab–trastuzumab–nab-paclitaxel (PERUSE) [82]		nr	2
	Tucatinib–trastuzumab–capecitabine [81]		45	5
	Tamoxifen (ABC) [30,31]		3 (lethargy)–18	1
	Anastrozole (ABC) [31,32]		1.2 (lethargy)–16 (asthenia)	0
	Letrozole (MONALEESA-2c) [30,31,83,84]		11–27	0–1
	Exemestane [31,85]		22–26	0
	Fulvestrant (CONFIRM, PALOMA-3c, MONARCH2c, and MONALEESA-3c) [86,87,88,89,90]		31–44	<1–3
	Lapatinib letrozole [91,92]		21–33 (ctrl: 17)	0–2 (ctrl: <1)
	Exemestane–Everolimus [85,93,94] BALLET: sub-analysis by ages (<70 vs. ≥70, non-comparative)		23 (asthenia)–50 29 vs. 21	2–4 6 vs. 3
	Palbociclib letrozole (PALOMA-1 and PALOMA-2) [83,95]		37–40 (ctrl: 23–28)	2–4 (ctrl: 1)
	Palbociclib fulvestrant (PALOMA-3) [88]		44 (ctrl: 31)	3 (ctrl: 1)
	Palbociclib-ET (pooled analysis PALOMA 1, 2, 3)			
	<65 [96]		40 (ctrl: 27)	2 (ctrl: 1)
	65–74 [96]		41 (ctrl: 28)	3 (ctrl: 0)
	≥75 [96]		37 (ctrl: 31)	6 (ctrl: 0)
	Ribociclib letrozole (MONALEESA-2, CompLEEment-1) [84,97]		23–42 (ctrl: 27)	1–2 (ctrl: 1)
	Ribociclib fulvestrant (MONALEESA-3) [90]		32 (ctrl: 33)	2 (ctrl: <1)
	Abemaciclib letrozole or anastrozole (MONARCH-3) [98]		40 (ctrl: 32; gr2+: 17)	2 (ctrl: 0)
	Abemaciclib fulvestrant (MONARCH-2) [89]		40 (ctrl: 27)	3 (ctrl: <1)
	Talazoparib (EMBRACA) [99]		50 (ctrl: 43)	2 (ctrl: 3)
	Olaparib (OlympiaAD) [100]		29 (ctrl: 23)	3 (ctrl: 1)

The colors refer to the higher reported frequency of grade 3 or more fatigue for each regimen: 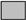: [30–40%]; 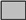: [20–30%]; 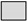: [10–20%]; 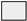: [5–10%]; 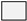: [0–5%]; 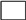: not reported. Data specific to older patients are underlined. Abbreviations: ctrl: control; ET: endocrine therapy; gr: grade; gr2+: grade 2 or more; nr: not reported.

**Table 2:** Fatigue events in the most common anticancer regimens for ovarian cancer. Abbreviations: AUC: area under the curve; ctrl: control; nr: not reported; PLD: pegylated liposomal doxorubicin.

Scheme	Treatment Regimen and Reference	Fatigue Any-Grade (%)	Fatigue Gr ≥ 3 (%)
First line	3w-carboplatin monotherapy (ICON3) ≥70 (EWOT-3, EWOC-1) [66,106]	nr 73	nr 8–15
	3w-cisplatin 75 mg/m^2^ paclitaxel 185 mg/m^2^ (AGO OVAR-3) [108]	nr	nr
	3w-cisplatin 75 mg/m^2^ paclitaxel 135 mg/m^2^ (GOG-158) [109]	nr	nr
	3w-carboplatin AUC6 paclitaxel 185 mg/m^2^ (AGO OVAR-3) [108]	nr	nr
	≥70 (AGO OVAR-3) [110]	nr	nr
	3w-carboplatin AUC7.5 paclitaxel 175 mg/m^2^ (GOG-158) [109]	nr	nr
	3w-carboplatin AUC5 paclitaxel 175 mg/m^2^ (MITO-2c) [107]	44	3
	≥70 and GVS ≥3 (EWOC-1) [106]	70	10
	3w-carboplatin AUC6 paclitaxel 175 mg/m^2^ (MITO-7) [111]	48	5
	3w-carboplatin AUC5 PLD 30 mg/m^2^ (MITO-2i) [107]	43	3
	3w-carboplatin AUC6 w-paclitaxel 80 mg/m^2^ (GOG262) [112]	nr	nr
	w-carboplatin AUC2 w-paclitaxel 60 mg/m^2^ (MITO-7) [111]	55	4
	Regimen specific to older patients 3w/4 carboplatin AUC2 + paclitaxel 60 mg/m^2^ (MITO-5, EWOC-1) (≥70) [107]	38	0
	≥70 and GVS ≥3 (EWOC-1) [106]	85	10
	15 mg/kg bevacizumab maintenance 3w-carboplatin AUC6 + paclitaxel 175 mg/m^2^ (GOG-218, PAOLAc) [113]	32	1
	7.5 mg/kg bevacizumab maintenance 3w-carboplatin AUC6 + paclitaxel 175 mg/m^2^ (ICON7) [114]	nr	nr
	Olaparib maintenance (SOLO1) [103]	63 (ctrl: 42)	4 (ctrl: 2)
	Niraparib maintenance (PRIMA) [104] ≥65 [115]	35 (ctrl: 30) nr	2 (ctrl: <1) nr
	Rucaparib maintenance		
	Olaparib bevacizumab maintenance (PAOLA) [113]	53 (ctrl: 32)	5 (ctrl: 1)
Platin-sensitive relapse	PLD/carboplatin [107]	43	3
	Paclitaxel/carboplatin [107]	44	3
	Gemcitabine/cisplatin [116]	28	2
	Trabectidin/PLD [117]	nr	6
	Bevacizumab 3w-carboplatine w-paclitaxel (OCTAVIA) [118]	nr	nr
	Bevacizumab carboplatin gemcitabine 1000 mg/m^2^ J1,J8 (OCEANS) [119]	nr	nr
	Olaparib maintenance (SOLO2) [120]	62 (ctrl: 37)	4 (ctrl: 2)
	Niraparib maintenance (NOVA) [121]	59 (ctrl: 41)	8 (ctrl: 1)
	<70 [122]	nr	8 (ctrl: 2)
	≥70 [122]	nr	8 (ctrl: 0)
	Rucaparib maintenance (ARIEL3) [123]	69 (ctrl: 44)	7 (ctrl: 3)
	Niraparib alone (w/o CT) (AVANOVA2) [124]	39	2
	Bevacizumab + niraparib w/o CT (AVANOVA2) [124]	40	6
Platin-resistant relapse	Paclitaxel 175 mg/m^2^/3 sem [125]	nr	nr
	Paclitaxel 175 mg/m^2^/3 sem [125]	nr	nr
	Paclitaxel 225 mg/m^2^/3 sem [125]	nr	nr
	Weekly paclitaxel	nr	nr
	3w-topotecan [126,127]	31–46	0–2
	w-topotecan [128,129]	32	2–22
	PLD [117,130,131]	22–44	1–6
	Gemcitabine [130]	nr (gr2: 36)	11
	Docetaxel [132]	nr	nr
	Bevacizumab + chemotherapy (AURELIA) [133]	nr	4 (ctrl: 10)

The colors refer to the higher reported frequency of grade 3 or more fatigue for each regimen: 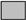: [30–40%]; 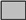: [20–30%]; 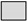: [10–20%]; 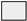: [5–10%]; 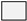: [0–5%]; 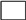: not reported. Data specific to older patients are underlined. Abbreviations: ctrl: control; gr: grade; gr2+: grade 2 or more; nr: not reported.

**Table 3:** Fatigue events in the most common anticancer regimens for prostate and urothelial cancers. Abbreviations: ADT: androgen deprivation therapy; ctrl: control; DDGc: dose dense with G-CSF; gr: grade; gr2+: grade 2 or over; LHRHa: LHRH analog; nr: not reported; (2/3)w (bi/three)-weekly.

Setting	Treatment Regimen and Reference	Fatigue Any-Grade (%)	Fatigue Gr ≥3 (%)
**Prostate cancers**			
Non metastatic castration resistant	ADT + apalutamide (SPARTAN) [136]	33 (ctrl: 14)	3 (ctrl: 1)
Metastatic castration sensitive	ADT [6] (LATITUDEc, STAMPEDEc, CHAARTEDc, TITANc, and SPARTANc) [136,137,138,139,140,141]	Any (FSS): 74% 14–20	Severe (FSS): 14% <1–4
	LHRHa:		
	Leuprolide [135,142]	6	nr
	Goselerin [143,144]	nr	nr
	Triptorelin [142]	nr	nr
	Degarelix (induction 240 mg/maintenance 80 mg)	3	0
	ADT + abiraterone acetate (LATITUDE*i* and STAMPEDE*i*) [137,138]	13 (ctrl: 14)	2 (ctr: 2)
	ADT + enzalutamide (ENZAMET and ARCHES) [139,140]	24 (ctrl: 20)	2–6 (ctrl: 1–2)
	ADT + apalutamide (TITAN and SPARTAN) [136,141]	20–32 (ctrl: 15–17)	1–2 (ctrl: <1–1)
	ADT + docetaxel 75 mg/m^2^/3w (GETUG-AFU-15, E3805, and STAMPEDE) [145,146]	74 (ctrl: 20)	4–7 (ctrl: 1–4)
Metastatic castration resistant	ADT + abiraterone acetate		
	After docetaxel (COU-AA-301) [147] <75 [148] ≥75 [148] In chemo-naive patients (COU-AA-302) [149]	44 (ctrl: 43) 47 (ctrl: 45) 48 (ctrl: 42) 39 (ctrl: 34)	8 (ctrl: 10) 8 (ctrl: 11) 13 (ctrl: 11) nr
	ADT + enzalutamide (STRIVE) [150]	38 (ctrl: 28)	5 (ctrl: 3)
	After docetaxel (AFFIRM) [151] At baseline [152] In chemo-naïve patients (PREVAIL) [153]	34–62 (ctrl: 29, gr2+: 9) 64 (gr2+: 9) 36 (ctrl: 26)	6 (ctrl: 7) 2 (ctrl: 2) nr
	3w-docetaxel 75 mg/m^2^/3w [154]	53	5
	w-docetaxel 30 mg/m^2^/w [154]	49	5
	2w-docetaxel 50 mg/m^2^ [155]	65 (cyc; ctrl: 51)	3 (cyc, ctrl: 3)
	Cabazitaxel 25 mg/m^2^/3w [156]	37	5
	Cabazitaxel 20 mg/m^2^/3w [157]	nr	nr
	Radium 223 chloride [158]	24 (plb: 24)	3 (plb: 6)
	Olaparib [159]	41 (ctrl: 32)	3 (ctrl: 5)
**Urothelial cancers**			
**1st line**	MVAC [160,161]	nr	24
	DDGc MVAC [161,162]	nr	nr
	Paclitaxel 225 mg/m^2^/carboplatin AUC6 [160]	nr	10
	Gemcitabine cisplatin [163]	nr	nr
	Gemcitabine weekly cisplatin [163]	nr	nr
	Gemcitabine carboplatin [163]	nr	nr
	Avelumab maintenance (JAVELIN Bladder 100) [164]	18 (ctrl: 7)	2 (ctrl: 1)
**2nd line**	Gemcitabine [163]	nr	nr
	Vinflunine [165]	50 (ctrl: 61)	19 (ctrl: 18)
	Pembrolizumab (KEYNOTE-045) [166]	14 (ctrl: 28)	1 (ctrl: 4)
**3rd line**	Enfortumab vedotin (EV201, EV301) [167,168]	31–50 (ctrl: 23)	6 (ctrl: 5)

The colors refer to the higher reported frequency of grade 3 or more fatigue for each regimen: 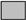: [30–40%]; 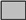: [20–30%]; 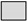: [10–20%]; 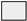: [5–10%]; 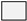: [0–5%]; 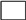: not reported. Data specific to older patients are underlined.

**Table 4:** Fatigue events in the most common anticancer regimens for colorectal cancers.

Scheme 3	Treatment Regimen and Reference	Fatigue Any-Grade (%)	Fatigue Gr ≥3 (%)
Adjuvant	5-FU/leucovorin (MOSAIC*c*) [172,173]	2–25	<1–2
	Capecitabine [173]	23	1
	FOLFOX (MOSAIC*i and* PETACC-8c) [172,174,175]	63	4–5
	XELOX/CAPOX 6mo (TOSCA, IDEA, SCOT, and HORG) [169,170,171]	SCOT: 90 (*p* = 0.022) TOSCA: 39 (asthenia)	SCOT: 8 IDEA: 5 (*p* = 0.0027) TOSCA: 4 (asthenia)
	XELOX/CAPOX 3mo (TOSCA, IDEA, SCOT, and HORG) [169,170,171]	SCOT: 86 (*p* = 0.022) TOSCA: 28 (asthenia, *p* < 0.0001)	SCOT: 8 IDEA: 3 (*p* = 0.0027) TOSCA: 1 (asthenia)
Metastatic	Capecitabine ≥70 (AVEX*c*) [176]	21–23 27	1 1
	FOLFOX [177,178]	42	0–6
	≥72 [179]	75	10
	XELOX [180]	62	9
	≥65 vs. < 65 [181]	64 vs. 63	22 vs. 19
	≥70 [182]	38	16
	2w-XELOX/CapeOX regimen specific to older patients (≥70) [183]	46	6
	FOCUS2 trial on older/frail patients not candidate for standard full-dose chemotherapy [184]:		
	80% FU 80% FOLFOX (OxFU) 80% Cap (Capecitabine) 80% CapOx Fluorouracil vs. capecitabin (FU/FOLFOX vs. Cap/CapOx) Addition of oxaliplatin (FOLFOX/CpOx vs. FU/Cap)	nr (lethargy ≥ gr2: 38) nr (lethargy ≥ gr2: 42) nr (lethargy ≥ gr2: 36) nr (lethargy ≥ gr2: 43) lethargy ≥ gr2: 40 vs. 39 lethargy ≥ gr2: 37 vs. 43	7 (lethargy) 9 (lethargy) 13 (lethargy) 15 (lethargy) 8 vs. 14 (lethargy, *p* = 0.06) 10 vs. 12 (lethargy; *p* = 0.88)
	FOLFIRI [185,186,187,188,189]	43–46	0–6
	XELIRI [190,191,192,193,194,195]	8–48 (≥gr2: 22%)	0–8
	CAPIRI [196]	nr	nr
	XELIRI weekly	nr	5
	XELIRI 2-weekly [197]	57	13
	FOLFOXIRI/FOLFIRINOX [186,189]	nr	6
	≥70 [198]	94	10
	XELOXIRI [199]	50	3
	COI 2-weekly [200]	nr	nr
	IFL 21d [201]	35	0–6
	IRINOX [202]	90	13
	Raltitrexed [203]	nr	5
	TOMOX [178,204,205]	35–53	4–16
	TOMIRI [205,206]	33–53	3–7
	Bevacizumab–FOLFIRI [189,207,208,209]	55–75	1–9
	Bevacizumab XELIRI [210,211]	67	7–17
	Bevacizumab–FOLFOX [177,209,212]	54–71	1–11
	Bevacizumab XELOX [213]	5	9
	≥70 [214]	62	16
	≥75 [214,215]	28–84	8
	Bevacizumab–FOLFOXIRI [189,207]	nr	12
	Bevacizumab capecitabine		
	≥70 (AVEX*i*) [176]	24	4
	≥70/PS = 2 [216]	67	13
	Aflibercept–FOLFIRI [217]	60	17
	Cetuximab [218]	nr	33 (ctrl: 26)
	Cetuximab–FOLFIRI [188,208]	49	<1–4
	Cetuximab XELIRI [191]	nr	5 (asthenia)
	Cetuximab–FOLFOX [219,220]	66	8
	Cetuximab XELOX [220]	nr	nr
	Cetuximab FOLFIRINOX [221]	nr	32
	Cetuximab-irinotecan [222,223]	34–75	3–5
	Panitumumab–FOLFIRI [224]	nr	nr
	Regorafenib [225]	47 (ctrl: 28)	9 (ctrl: 5)
	≥70 (REGOLD) [226]	90	45
	TAS-102 (trifluridine–tipiracil) (RECOURSE) [227,228]	35–85 (ctrl: 23)	4–11 (ctrl: 6)
	Bevacizumab-TAS-102 (C-TASK FORCE) [228,229]	24–85	0–7
	Encorafenib cetuximab (BEACON) [230]	30 (ctrl: 27)	4 (ctrl: 4)
	Pembrolizumab (KEYNOTE 177) [231]	38 (ctrl: 50)	4 (ctrl: 9)

The colors refer to the higher reported frequency of grade 3 or more fatigue for each regimen: 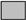: [30–40%]; 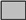: [20–30%]; 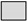: [10–20%]; 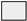: [5–10%]; 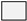: [0–5%]; 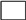: not reported. Data specific to older patients are underlined.

**Table 5:** Fatigue events in the most common anticancer regimens for lung cancers.

Setting	Treatment Regimen and Reference	Fatigue Any-Grade (%)	Fatigue Gr3 (%)
Non-small cell lung cancer	Docetaxel [238]	27	4
	Docetaxel/carboplatin [233]	nr	10–16 16 (weakness) 10 (asthenia)
	Etoposide/cisplatin [239]	nr	nr
	Paclitaxel/cisplatin [232,233]	nr	9 (lethargy)- 15 (weakness)
	Docetaxel/cisplatin [232,233,240]	nr	7–16
	Vinorelbine/cisplatin [233]	nr	9
	Paclitaxel/carboplatin [232,233]	nr	9–15
	Carboplatin paclitaxel regimen specific to older patients (3w-carboplatin AUC6, w-paclitaxel 90mg/m^2^) (≥70) [241]	nr	10 (asthenia)
	Pemetrexed/cisplatin [233]	nr	7
	Carboplatin/pemetrexed [242] PCb5 (Carboplatin AUC5) PCb6 (Carboplatin AUC6)	11–48 11 48	0–3 0 3
	Pembrolizumab/pemetrexed/cisplatin or carboplatin [236]	41	6
	Gemcitabine/cisplatin [232,233,240]	nr	8–40
	Gemcitabine/carboplatin [233]	nr	40
	Bevacizumab carboplatin/paclitaxel [234,235]	20–45	3–5
	Bevacizumab atezolizumab carboplatin/paclitaxel [235]	22	3
	Bevacizumab carboplatin/pemetrexed [234]	53	11
	Nivolumab 10mg/kg + gemcitabine/cisplatin [237]	67	0
	Nivolumab 10mg/kg + pemetrexed/cisplatin [237]	80	7
	Nivolumab 10mg/kg + paclitaxel/carboplatin [237]	67	13
	Nivolumab 5mg/kg + paclitaxel/carboplatin [237]	71	0
	Gemcitabine/docetaxel ≥70 [243]	nr 48	nr 6
	Gemcitabine (maintenance) [244] ≥70 [243]	nr 31	nr 6
	Pemetrexed (maintenance) [245]	24	5
	Bevacizumab pemetrexed (maintenance) [234]	49	3
	Bevacizumab (maintenance) [234]	62	12
	Erlotinib [233]	3	0
	Crizotinib [246]	27	2
	Ceritinib [247]	47	5
	Gefitinib [233]	nr	3
	Nintedanib-docetaxel [238]	30	6
Small cell lung cancer	ACE [248]	nr	13
	Topotecan [249,250]	26	4
	Topotecan/paclitaxel [251]	nr	22
	ICE [252,253,254]	nr	nr
	wICE [252]	nr	nr
	VICE [255,256]	66	nr
	DDG ACE [257]	nr	nr
	DDG ICE [253]	nr	nr
	DDG CAV → PE [258]	nr	nr
	Paclitaxel carboplatin topotecan [259]	nr	12
	CAV [249]	34	nr
	Etoposide/cisplatin [260,261,262,263]	11	<1–2
	Etoposide/carboplatin [263,264]	11	<1
	Irinotecan/carboplatin [264,265]	nr	nr
	Irinotecan/cisplatin [261]	nr	nr
	Ipilimumab-Etoposide/cisplatin [263]	13	2
	Topotecan/cisplatin [262,266]	13	1–3
	Docetaxel/gemcitabine [267] Age > 65 or PS=2 [268]	nr nr	nr 25
	Irinotecan/gemcitabine [269]	nr	9–15
	CODE [270,271]	nr	nr
	CAV → PE [258,270,271]	nr	nr
	Paclitaxel/carboplatin [248] consolidation after CT-RT [272]	nr nr	9 15
	Nab P/C 3w [273]	nr	1
	Nab-P/C w [273]	nr	0
	wPaclitaxel 80mg/m^2^/sem [274]	27	1
	Gemcitabine [275,276]	nr	nr

The colors refer, for each regimen, to the higher reported frequency of grade 3 or more fatigue: 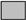: [30–40%]; 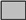: [20–30%]; 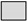: [10–20%]; 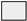: [5–10%]; 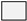: [0–5%]; 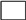: not reported. Data specific to older patients are underlined.

**Table 6:** Fatigue events in the most common anticancer regimens for non-Hodgkin’s and follicular lymphoma.

Setting	Treatment Regimen and Reference	Fatigue Any-Grade (%)	Fatigue Gr3 (%)
Diffuse large B-cell lymphoma	CHOP-21 [286] CHOP: data specific to older patients (60–80) [280,281,282]	nr nr	nr nr
	DHAP [287]	nr	nr
	ESHAP [288,289,290]	nr	nr
	CNOP [286]	nr	nr
	ACOD [291]	3	0
	Fludarabine/mitoxantrone [292]	nr	nr
	R-CHOP-21 [277,278]	27–46	3
	R-CHOP: data specific to older patients (>60) [280,281,282,284,293,294]	49	0
	R-CHOP–Lenalinomide maintenance: data specific to older patients (58–80) [295]	nr	nr
	R-miniCHOP regimen specific to older patients (≥80) [283]	nr	nr
	R-COMP [284,296]: data specific to older patients	53	7
	R-mini-CHP: regimen specific to older patients (≥80) [297]	nr	nr
	R-mini-CEOP regimen specific to “frail” patients (64–84 years) [298]	nr	nr
	R-CVP [277]	53	<1
	R-mini-CVP regimen specific to older patients (≥80) [285]	58	47
	DA-EPOCH-R [299]	nr	nr
	R-pola-CHP 21 [278]	26	1
	R-DHAP [279]	nr	9
	R-GemOx [300]	nr	nr
	Lenalinomide–rituximab [301,302,303]	nr	13–23
	Lenalinomide [302,303,304]	34	7–9
	Tafasitamab–lenalidomide (L-MIND) [305]	15	2
	CAR-T Lisocabtagene maraleucel (TRANSCEND NHL 001) [306]	44	1
	CAR-T Axicabtagene ciloleucel (ZUMA-1) [307]	51	2
	CAR-T Tisagenlecleucel (JULIET) [308]	25	6
	CAR-T real life experience USA [309]	nr	nr
	France [310]	nr	nr
Indolent lymphomas	Rituximab [311]	22	nr
	Bendamustine-rituximab (BRIGHT) [277]	44–58	3–4
	R-CHOP (FOLL05 and BRIGHT) [277]	46	2
	R-CVP (FOLL05 and BRIGHT) [277]	53	<1
	R-FM (FOLL05) [312]	nr	nr
	Rituximab-chemotherapy [313]	37	nr
	G-CHOP (GALLIUM) [314]	16	nr
	G-CVP (GALLIUM) [314]	57	nr
	G-Bendamustine (GALLIUM) [314]	44	nr
	Obinutuzumab-chemotherapy [313]	36	nr
	Revlimid–lenalidomide (CALGB 50803) [315]	78	6

The colors refer to the higher reported frequency of grade 3 or more fatigue for each regimen: 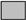: [30–40%]; 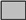: [20–30%]; 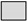: [10–20%]; 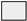: [5–10%]; 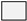: [0–5%]; 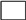: not reported. Data specific to older patients are underlined.

## Data Availability

Not applicable.
